# Refugees’ Opinions about Healthcare Services: A Case of Turkey

**DOI:** 10.3390/healthcare9050490

**Published:** 2021-04-21

**Authors:** Dilaver Tengilimoğlu, Aysu Zekioğlu, Fatih Budak, Hüseyin Eriş, Mustafa Younis

**Affiliations:** 1 Management Department, Faculty of Management, Atilim University, 06530 Ankara, Turkey; dilaver.tengilimoglu@atilim.edu.tr; 2 Health Management Department, Faculty of Health Sciences, Trakya University, 22100 Edirne, Turkey; 3 Health Management Department, Faculty of Health Sciences, Kilis 7 Aralık University, 79000 Kilis, Turkey; fbudak@kilis.edu.tr; 4 Medical Documentation, Vocational School of Health, Harran University, 63000 Şanlıurfa, Turkey; erisharran@hotmail.com; 5 College of Health Sciences, Jackson State University, Jackson, MS 39217, USA; younis99@gmail.com

**Keywords:** asylum seeker, refugee, migration, health services, Turkey

## Abstract

Background: Migration is one of the most important social events in human history. In recent years, Turkey hosted a high number of asylum seekers and refugees, primarily because of continuing wars and radical social changes in the Middle East. Methods: Using a random sampling method, Syrian refugees aged 18 and over, who can communicate in Turkish, were reached via personal contact and a total of 714 refugees participated in the study voluntarily. Results: Turkey has mounted with some success and to point out that even though participating refugees in both provinces are young and healthy, almost 50% have bad or worse health status, 61% have chronic diseases, and 55% need regular medication. Participating refugees living in Şanlıurfa stated that ‘Hospitals are very clean and tidy.’ (3.80 ± 0.80). The answers given to the following statements had the highest mean for the participating refugees living in Kilis; ‘Hospitals are clean and tidy.’ (3.22 ± 1.25). Conclusion: Due to financial and human resource deficiencies, there are problems in providing preventive and therapeutic health services, especially to refugees living outside the refugee camps in bad conditions. It is important that refugees are encouraged to apply to family health and community health centers in this context.

## 1. Introduction

Human migration is one of the most significant social events experienced by societies throughout human history. Along with the circumstances that cause migration, migration’s social and economic consequences have also been complex in recent years [1]. In addition to the camps, security, education, and employment problems faced by immigrants, asylum seekers, or refugees, the most significant constraint is health and access to healthcare services. It is observed that people from different countries and cultures are negatively affected by conditions such as poverty, lack of social support, ethnic and religious discrimination in the host country [2]. Besides, some of the pre-migration and post-migration risk factors create vulnerable populations. During the pre-migration process and detention, the traumas experienced by people in their own countries, the length of the asylum procedure, language barriers, and the lack of knowledge about the new health system in the host country also cause health-related problems that impact the whole society [3].

Although countries hosting migrants, asylum seekers, and refugees have developed various policies and practices regarding public services, there are still limitations in access to healthcare services and social security. For example, in England, while making arrangements for refugees to receive healthcare services in the national health program, refugees have encountered problems in accessing services due to various factors, including the language barrier [4]. The ‘45 days rule’ was first introduced in 2009 for refugees who could not qualify for immigration status in Australia. While refugees benefited from healthcare services through universal health insurance for the first 45 days, they could not access health care services after 45 days [5]. The situation is similar in France [4]. Refugees live in reception centers in the Netherlands. Those with immigrant status can directly access primary health care services. When refugees want to receive health services, they must first be referred to the nurses at the community health center [6]. In Ireland, while the application is processed, refugees receive an allowance of approximately €19 per week. They are eligible to take literacy and English language courses for adults while staying in a center where all three meals are served. Moreover, asylum seekers and refugees who are entitled to receive a medical card due to the Legal Aid test have the right to benefit from free healthcare services, including access to a general practitioner, prescription of drugs, and psychology services [7].

In recent years, Turkey has become one of the leading countries hosting high numbers of asylum seekers and refugees, mainly due to the current war and radical social changes experienced in the Middle East. As of 2019, 3,674,588 Syrian emigrants and 370,400 emigrants from other countries as of 2018 were placed under temporary protection in Turkey [8,9]. In Turkey, Kilis is the province where most of the population comprises Syrian refugees compared to the local population (81.58%). Hatay (27.32%), Gaziantep (22.24%), and Şanlıurfa (21.04%) are other provinces in Turkey where refugees are mostly residing [10]. As a result of this intense migration flow of Syrian refugees, who now represent approximately 4.40% of Turkey’s overall population, the Ministry of Interior and Directorate General of Migration Management started to determine various strategic objectives to implement in health, education, social integration, communication, and food [11]. According to the report titled ‘Health Status Survey of Syrian Refugees In Turkey’, conducted by the Ministry of Health, The Disaster and Emergency Management Presidency (AFAD), and World Health Organization, there were 5760 Syrian refugees in 2016. It was found that 34% of the Syrians participating in the study used tobacco products, and 37.2% did not consume vegetables and added salt before starting to eat. In the same report, 6.4% of the participants had a history of cardiovascular system disease, 7.2% of women between the ages of 18 and 69 were diagnosed with cervical cancer, 4.1% were diagnosed with high blood sugar in the last 12 months, and 3 to 5 risk factors were more common in men than in women [12]. In addition to the effect of genetic factors, living and working conditions, especially in harmful environments outside the camps, increase the need for refugees and asylum seekers to have access to healthcare services. Even though Turkey provides refugees and asylum seekers with healthcare services, they still have difficulties accessing healthcare services because of cultural and language barriers, insufficient information about the Turkish healthcare system, and lack of insurance and income to cover their healthcare expenses. In this sense, it is essential to access health services by asylum seekers and refugees. Therefore, this study aims to examine the views about the healthcare services of the refugees living in Kilis and Şanlıurfa, where most of the population comprises Syrian refugees compared to the local people, to offer suggestions based on the results.

## 2. Materials and Methods

### 2.1. Universe-Sample

According to the data from AFAD, among the provinces with the highest number of refugees in Turkey, Şanlıurfa ranks second with 424,331 refugees, and Kilis ranks the seventh with 125,668 refugees. The study focus was on Syrian refugees living in Şanlıurfa and Kilis as of May 2019. The study sample comprised 714 Syrian refugees aged 18 and over who could communicate in Turkish and were reached using a random sampling method. Refugees, who were reached via personal contacts, were numbered according to the place they worked in and the neighborhoods they lived in. A random number was chosen from this list, and the participants were interviewed.

### 2.2. Data Collection Tool

The research is a cross-sectional field study. A questionnaire developed by the researchers was used in the study. The questionnaire form is composed of two parts. The first part included questions about the demographic data of the refugees who participated in the study. In the second part, there was a 5-point Likert-type scale designed to measure refugees’ opinions and suggestions about healthcare service delivery. A 14-question 5-point Likert-type scale had items for determining refugees’ views on healthcare services. Cronbach Alpha reliability coefficient, which was calculated to demonstrate the reliability of the current study, was 0.712. Ethical committee approval was obtained from Trakya University Social Sciences and Humanities Research and Ethics Committee, Number: 2019/02.

A total of 10 interviewers (5 were Şanlıurfa, 5 were Kilis) were employed to administer the interviews. Supervisors (co-authors from Şanlıurfa and Kilis) informed the interviewers about the purpose of the study, the content of the questionnaire, and the ethical conditions. Participants who have met eligibility and inclusion criteria (aged 18 and over, can communicate in Turkish) were reached via personal contact. Participants were included in the study voluntarily, and participants did not receive any payments or compensations for participating in the study.

### 2.3. Data Analysis

SPSS 22.0 (IBM, Armonk, NY, USA) was used to analyze the data. Demographic data were calculated by percentage and frequency. The Kolmogorov Smirnov test was administered to see whether the scale had a normal distribution. The *t*-test and ANOVA were used to analyze the data according to the normality test results.

## 3. Results

When the demographic data of the refugees participating in the study were examined, around half of the participants living in Şanlıurfa were female, most of them were married. Most of the refugees living in Kilis who participated in the research were female, most were single. The majority of the participants living in both provinces did not have any stable income (Table 1).

When the answers to the questions about health status and access to healthcare services were analyzed, it was found that most of the participants in both provinces stated that they went to the state hospital when they needed healthcare services. It has been observed that because of out-of-pocket payments for healthcare services provided in private hospitals, they are directed to state hospitals where free-of-charge services are provided. About 46 percent rated their health status as bad or worse, and 56 percent reported having chronic diseases, and over half required medications. The majority of the participants in both provinces defined their health as ‘good’, probably because the average age of the participants was low (29.2 ± 11.6) (Table 2).

In recent years, with the health reform in Turkey, new hospital buildings were built, which reduced the waiting time. This situation is also reflected in the current study results. In terms of the responses of the participating refugees living in both provinces, they stated that the hospitals were clean and tidy (3.55 ± 1.05). They also remarked that the waiting time for examination was not too long in the laboratory/radiology units (2.60 ± 1.28) (Table 3).

Non-parametric tests were used to analyze the data gathered from the 14-question 5-point Likert-type scale because the scale showed normal distribution (*p* > 0.05) after running the Kolmogorov Smirnov normality test.

*T*-test for independent groups was used to investigate whether there were significant differences between the views of the refugees about the healthcare services in the provinces where they reside. According to the analysis results, there were no significant differences between provinces in terms of refugees’ views on healthcare services (*p* > 0.05) (Table 4).

Similarly, a *t*-test for independent groups was used to see whether there were any differences between the genders of the refugees about the healthcare services. According to the results, no significant differences were found between genders regarding views on healthcare services (*p* > 0.05) (Table 5).

One-way analysis of variance (ANOVA) was run to investigate whether there were any differences between the views of the refugees participating in the study about healthcare services in terms of their education level/background. According to the analysis results, there were no significant differences between the education levels in terms of views on healthcare services (*p* > 0.05) (Table 6).

## 4. Discussion

Laws, regulations guarantee health services provided for refugees, and circulars such as AFAD Circular, the Directive on Migrant Health Centers/Units, and the Circular on Health Services Provided for People under Temporary Protection in Turkey. With the protocol signed between the Ministry of Health and AFAD, refugees have access to healthcare services 24/7 in centers called ‘temporary health facilities’ in camps with the support of an interpreter and staff healthcare professionals. AFAD covers health, medication, and medical equipment expenses of the refugees staying in the camps. Refugees under temporary protection receive health care services at family health centers and migrant health centers established by the SIHHAT project. In these centers, healthcare services, such as vaccination, screening, and psycho-social support, are offered free of charge. Refugees cannot go directly to the state or private hospitals, except for an emergency. The primary care center should give an official referral for hospital admissions. According to the legislation, when refugees under temporary protection ID receive healthcare services, the invoice is issued on behalf of the Governorship. The appropriate invoice is paid by the Governorship [13].

Turkey is making efforts in financial and human resources to provide healthcare services for refugees and trying to use the most effective ways to eliminate the limitations experienced in access to health care [14]. Accordingly, it is a crucial step to give an ear to the satisfaction levels of the refugees with the healthcare services and offer solutions first-hand. According to ‘Demographic Outlook of Syrians in Turkey, Living Conditions and Future Prospects of the Oriented Field Survey’ conducted in 2017 by AFAD [15], refugees’ access to healthcare services was 97.2% in camps and 62.9% outside of the camps. Moreover, refugees living in camps stated that they were satisfied with healthcare services in Turkey (48.8%). Those residing outside camps also stated that they were satisfied with the healthcare services (58.8%). In the present study, refugees living in Şanlıurfa and Kilis responded similarly to the survey, and they stated that the hospitals were clean and tidy. The physicians and other healthcare professionals were kind and helpful.

In studies conducted with refugees in different countries, it is seen that the common problem is the language barrier for access to healthcare services [16,17,18,19]. In our study, the participants living in Şanlıurfa and Kilis stated that they sometimes had difficulty expressing themselves to healthcare professionals. Additionally, they sometimes had difficulty in understanding what physicians and healthcare professionals were telling them. Language and cultural barriers had a negative effect on access to health care. In this context, it is necessary to expand interpreter services at hospitals in provinces where the number of refugees is high.

The study results show that one of the crucial issues in refugees’ healthcare services is the lack of social security and out-of-pocket payments [20,21]. In the present study, participants living in Şanlıurfa and Kilis stated that they sometimes had difficulties covering their health expenses due to social security problems. As refugees refrain from demanding health services due to financial constraints and lack of insurance, the necessary infrastructure should be established for refugees outside the camps to go to the social security institution and obtain the appropriate insurance status.

The refugees living in Şanlıurfa and Kilis stated that they mostly thought that the physicians who examined them did not spare enough time for them (mean: 2.88). Physicians and other health care professionals did not provide them with adequate care because they were immigrants/refugees (mean: 3.19). However, it is believed that this situation may occur due to the high workload of physicians.

## 5. Study Limitations

Firstly, one of the limitations of this research is that it was conducted only with refugees who can speak Turkish. Secondly, the study was limited to only two provinces. The third limitation is that the average age of participants was low, so they may not need as many healthcare services. Similar studies should be carried out by conducting qualitative interviews with refugees who do not speak Turkish, the elderly, and individuals with chronic disease who use healthcare services more.

## 6. Conclusions

Access to health care is one of the most fundamental human rights. Migration movements due to war, terrorist incidents, and natural disasters have a significant effect on individuals’ health. Therefore, individuals encounter problems accessing healthcare services. People who can easily access healthcare services in their own countries cannot receive similar health care services due to lack of social security, lack of knowledge, language, and cultural barriers. Especially refugees confront problems when they settle in a host country with refugee status. The challenges as a whole pose real threats to both individual and public health. Turkey puts great effort into providing health care for all while hosting refugees from many countries. However, due to limited financial and human resources, there are still some obstacles in providing preventive and curative health services to undocumented refugees, especially those living outside the camps. We hope that such descriptive studies with refugees living in various provinces of Turkey will provide efficient tools and approaches for future management and planning activities of essential services, including healthcare.

## Figures and Tables

**Table 1:** Descriptive data of participants.

**Variables**		**ŞanlıUrfa (*n* = 414)**		**Kilis (*n* = 300)**		**General (*n* = 714)**	
		**Number**	**%**	**Number**	**%**	**Number**	**%**
Gender	Female	219	52.9	175	58.3	394	55.3
	Male	195	47.1	125	41.7	320	44.7
Marital Status	Married	237	57.2	95	31.6	332	44.4
	Single	177	42.8	205	68.4	382	55.6
Education Level	No education	72	17.4	6	2.0	78	9.5
	Primary school	36	8.7	66	22.0	102	15.4
	High School	98	23.7	110	36.7	208	30.2
	Associate Degree	74	17.9	10	3.3	84	10.6
	Bachelor	120	29.0	97	32.3	217	30.7
	Postgraduate	14	3.4	11	3.7	25	3.6
Age (Year) (Mean ± SD)		33.21 ± 13.9		25.12 ± 9.23		29.2 ± 11.6	
		**ŞanlıUrfa (*n* = 414)**		**Kilis (*n* = 300)**			
		**Number**	**%**	**Number**	**%**		
Do you work in any job?	Yes	255	61.6	88	29.4		
	No	159	38.4	212	70.6		
For how many years have you lived in Turkey?	Less than 1 year	126	30.5	15	5.0		
	1–4 years	239	57.7	117	39.0		
	5–9 years	49	11.8	163	54.3		
	More than 10 years	-	-	5	1.7		
Income	No Income	183	44.2	128	42.7		
	Less than 500 TL	79	19.1	61	20.3		
	501–1000 TL	55	13.3	55	18.3		
	1001–1500 TL	45	10.9	20	6.7		
	1501–2000 TL	27	6.5	21	7.0		
	More than 2000 TL	25	6.0	15	5.0		
How many people live in your house? (Mean ± SD)		4 ± 2		5 ± 2			

**Table 2:** Questions about health status and access to healthcare services.

		ŞanlıUrfa (*n* = 414)		Kilis (*n* = 300)	
		Number	%	Number	%
How can you describe your health status?	Excellent	28	6.8	71	23.6
	Good	196	47.3	191	63.7
	Bad	160	38.6	35	11.7
	Worse	30	7.3	3	1.0
Do you have any chronic diseases?	Yes	231	55.8	20	6.7
	No	183	44.2	280	93.3
Do you use any regular medication?	Yes	214	51.7	12	4.0
	No	200	48.3	288	96.0
Which institution do you receive health care from?	Primary care center	68	16.4	31	10.3
	State hospital	320	77.3	249	83.0
	Private hospital	26	6.3	20	6.7
Which healthcare services have you received in the last 6 months?	Outpatient services	174	42.0	108	36.0
	Surgery	33	8.0	30	10.0
	Inpatient treatment (medication)	21	5.1	11	3.7
	Emergency	56	13.5	47	15.6
	Dental	38	9.2	45	15.0
	Vaccination	3	0.7	3	1.0
	Prescribed medicine	41	9.9	41	13.7
	Check-up	48	11.6	15	5.0

**Table 3:** The refugees’ views on healthcare services.

	Şanlı Urfa (Mean ± SD)	Kilis (Mean ± SD)	General (Mean ± SD)
Waiting time for examination is too long in the laboratory/radiology unit.	2.49 ± 1.16	2.74 ± 1.41	2.60 ± 1.28
Hospitals are very clean and tidy.	3.80 ± 0.80	3.22 ± 1.25	3.55 ± 1.05
Physicians and other health professionals are kind and helpful.	3.18 ± 0.90	3.03 ± 1.12	3.11 ± 1.00
The physician who examined me spares me enough time.	2.85 ± 1.03	2.92 ± 1.19	2.88 ± 1.10
I have difficulty expressing myself to physicians and other healthcare professionals.	2.82 ± 1.03	2.86 ± 1.10	2.84 ± 1.06
I find it difficult to understand what my physician and other health care professionals are telling me	2.71 ± 0.91	2.89 ± 1.02	2.79 ± 0.96
Due to the lack of social security, I have difficulty in covering my health expenses.	2.93 ± 1.24	2.93 ± 1.08	2.93 ± 1.17
When I go to the hospital for examination, I am informed about the processes by the officials.	3.07 ± 1.01	3.11 ± 1.15	3.08 ± 1.07
Physicians and other healthcare professionals take care of my privacy.	2.95 ± 1.18	2.99 ± 1.20	2.97 ± 1.19
I ask the physician to prescribe the drugs which I want.	2.71 ± 1.04	2.85 ± 1.117	2.77 ± 1.09
I constantly have health problems because I have problems in terms of nutrition, shelter, and hygiene	2.71 ± 0.98	2.73 ± 1.08	2.72 ± 1.02
I trust the knowledge of physicians and other health care professionals and follow the treatment protocol they instruct	3.13 ± 1.09	3.13 ± 1.16	3.13 ± 1.12
I think that I am not provided with sufficient care by physicians and other health care professionals because I am an immigrant/refugee	3.32 ± 1.34	3.02 ± 1.22	3.19 ± 1.30
I avoid giving consent because I do not understand what is written in the information and consent forms.	2.80 ± 1.22	2.70 ± 1.14	2.76 ± 1.19

**Table 4:** The comparison of the opinions of refugees’ on healthcare services by provinces.

Group	N	Average	Sd	*t*	df	*p*
Şanlı Urfa	414	2.96	0.43	−0.640	561.9	0.522
Kilis	300	2.93	0.52			

**Table 5:** The comparison of the opinions of refugees’ on healthcare services by gender.

Group	*N*	Average	Sd	*t*	df	*p*
Female	394	2.95	0.50	0.074	710.7	0.941
Male	320	2.95	0.42			

**Table 6:** Correlations between the opinions of refugees’ on refugee-oriented healthcare services and education levels.

Group	*N*	Average	Sd	F	*p*
No education	78	2.87	0.45	0.491	0.783
Primary school	102	2.97	0.53		
High School	208	2.96	0.54		
Associate Degree	84	2.93	0.29		
Bachelor	217	2.95	0.43		
Post graduate	25	2.91	0.46		
